# The Feasibility and Acceptability of an App-Based Intervention Aimed at Improving Maternal Health Literacy Regarding Infant Play and Development: Mixed Methods Study

**DOI:** 10.2196/76517

**Published:** 2025-09-04

**Authors:** Fiona Bennin, Shane A Norris, Alessandra Prioreschi

**Affiliations:** 1 SA MRC/ Wits Developmental Pathways for Health Research Unit, Department of Paediatrics Faculty of Health Sciences, School of Clinical Medicine University of the Witwatersrand Johannesburg South Africa; 2 School of Human Development and Health University of Southampton Southampton United Kingdom

**Keywords:** acceptability, feasibility, mobile health, mHealth, maternal health literacy, mothers, infants, play and development

## Abstract

**Background:**

Allowing infants access to unstructured, unrestricted play in their home environment is imperative for increasing healthy movement behaviors and, therefore, developmental outcomes. Interventions should equip mothers to provide opportunities for infant play as early as possible. Evaluating such interventions is necessary to understand the feasibility for scale-up and implementation in specific contexts. Furthermore, the appropriateness and relevance of standardized outcome measures in different ethnic and socioeconomic contexts should be determined to ensure validity.

**Objective:**

This study aimed to (1) test the feasibility and acceptability of an intervention aimed at improving maternal health literacy regarding infant play and development and (2) determine participants’ understanding of the study outcome questionnaires.

**Methods:**

This mixed methods study was nested within the Play Love and You (PLAY) study, a randomized controlled trial (PACTR202202747620052) designed to promote infant development. Mothers assigned to the PLAY study intervention arm at 6 months post partum (n=68) received telephone or in-person check-ins and assessments every 2 months and health literacy intervention content and resources (videos and infographics) delivered via a mobile app every week. Feasibility was assessed by monitoring appointment attendance (adherence) and frequency of access to the content via the app. Acceptability was explored using a questionnaire and 2 participant focus group discussions (FGDs) at the end of the study (12 months post partum). The FGDs also included questions exploring the participants’ understanding of the 4 study outcome measure questionnaires administered at both 6 and 12 months.

**Results:**

In total, 68 participants were enrolled in the study at 6 months, of whom 17 (25%) attended the FGDs (n=8, 47% in FGD 1 and n=9, 53% in FGD 2). A total of 79% (49/62) of the participants completed the acceptability questionnaire. The health literacy content was found to be highly acceptable based on qualitative and quantitative data. Most acceptability questions had 98% (48/49) positive answers. Participants enjoyed learning about active infant play and developmental milestones and how to make recycled toys. Over 80% of participants (62/68, 91%) attended the 12-month exit appointment. Most of the participants (47/62, 76%) could access the intervention content over the 12 months of the PLAY study, and of those, 60% (28/47) looked at content more than once a week, and 11% (5/47) did so every day. Less than a quarter (10/47, 21%) only looked at the content sporadically. Access was impacted by technical difficulties attributed to using inconsistent external service providers.

**Conclusions:**

This study was found to be acceptable to participants and feasible in this setting. The high acceptability of the intervention content and belief that other mothers would benefit from it suggests potential for effectiveness in similar communities. However, the feasibility of app-based interventions relies on consistent and low-cost management of digital tools in low-resource settings.

## Introduction

### Background

Due to the importance and vulnerability of the first 1000 days of an infant’s life, it is necessary to start developing a range of healthy behaviors from birth [1]. The numerous negative side effects of an inactive, sedentary lifestyle can take years to present; however, there is evidence of an association between increased physical activity in infants (aged 1-12 months) and improved motor and cognitive development, as well as adiposity in infancy and later life [2,3]. In the early months of life, allowing infants access to unstructured, unrestricted play in their home environment is imperative for development and for encouraging increased physical activity [4-6]. Therefore, it is imperative to focus interventions on these formative months to encourage healthy movement behaviors as early as possible.

Previous research suggests that improving an individual’s health literacy has potential to positively impact their self-efficacy, resulting in a positive effect on their health outcomes [7]. Similarly, when mothers are provided with the skills required to understand and implement parenting-related information, this can increase their motivation levels and ability to cope with their infants’ demands, resulting in favorable infant health outcomes [8]. Therefore, it has been hypothesized that increasing caregiver health literacy is key to caregivers developing a deeper understanding of the importance of play and development and increased self-efficacy and motivation, resulting in greater participation in play in the early months and the years to follow.

In real-world settings, determining the acceptability and feasibility of an intervention is important to understand the practicality and suitability of an intervention for scale-up and implementation in specific contexts [9]. Feasibility is usually measured retrospectively to provide an explanation as to why certain aspects of the study were a failure or a success [9]. Specific indicators that can be used to measure feasibility include retention (how many participants were retained in the study), intervention fidelity (the extent to which an intervention was implemented as designed), acceptability (how well the intervention was received by the target group), adherence (the extent to which the individuals followed the intervention as prescribed), recruitment methods, and user engagement [10]. Furthermore, certain outcome measures developed in higher-income countries may not be culturally appropriate to certain ethnic and socioeconomic groups in other settings, which makes it important to determine whether the measures used in a study are appropriate, relevant, and understood by the target group [10].

With the increase in access to mobile phones in low- and middle-income countries, there has been an increased potential to encourage positive health behavior change and outcomes at a community level making use of digital interventions [11]. Conversely, there are significant resource barriers to implementing mobile health (mHealth) interventions in low-resource settings that impact the feasibility of these interventions. Barriers include a lack of electricity to charge devices (due to power outages at health facilities), limitations in hardware and software requirements, and limited access to affordable data or Wi-Fi [12-14]. Furthermore, when end users can access digital information, some individuals do not have the health literacy or language skills needed to understand and use health information appropriately [15,16].

However, there are certain facilitators that aid in intervention feasibility. To overcome challenges with understanding health information, studies conducted in similar settings suggest using simple, lay language together with images, illustrations, and interactive content and including oral explanations for those who cannot read [15,17]. Health content creators should also use local languages to convey health information and have a good understanding of the local context [15]. According to Sekhon et al [18] (and the definition used in this study), for an intervention to be acceptable, individuals need to like the content, and it should fit with their value system and not take up too much time or take the place of other important daily activities. The intervention should also be coherent and perceived to be effective while improving self-efficacy [18].

### Objectives

The Play Love and You (PLAY) study is a randomized controlled trial (RCT) of an intervention designed to promote infant development through encouraging maternal self-efficacy using behavioral feedback and supportive microinterventions [19]. This study was nested within the PLAY study and fell under one of its secondary objectives, which was to test the efficacy, feasibility, and acceptability of providing intervention content and increasing opportunities for early learning through play to promote health literacy in mothers (in a low-resource setting) [19]. Findings from this study were to be used to guide the development of a high-fidelity intervention implemented at scale.

The specific objectives of this study were as follows:

1. To test the feasibility and acceptability of an intervention aimed at improving maternal health literacy regarding infant play and development
2. To determine participants’ understanding of the study outcome questionnaires’ content considering the context in which they live

## Methods

### Study Setting and Participants

The study described in this paper is a substudy of a larger RCT called the PLAY study. As per the PLAY study protocol, mother-infant pairs were recruited to the study within a month following delivery from 2 community clinics, Lilian Ngoyi and Chiawelo. Both clinics are primary clinics linked to Chris Hani Baragwanath Hospital in Soweto, South Africa. They both have midwife obstetrics units as well as postnatal clinics, which provide antenatal and postnatal care, respectively [19]. Soweto is an urban *township* located in the southwest of Johannesburg, with a population of approximately 1.9 million people [20]. As of 2023, approximately 9.9% of the Soweto population comprised children aged <6 years [20]. Participants were screened and enrolled in the study at their postnatal checkup 2 days after birth. Mothers were eligible for recruitment if (1) they were aged ≥18 years with an infant aged <1 month (2) of whom they were the primary caregiver and (3) were living in Soweto for the duration of the study. Mothers with both primigravida or multigravida birth experiences were included in the study. Participants were ineligible if infants were born before term. Each pair was individually sequentially randomized into either a control or intervention group using simple 1:1 randomization generated by Stata (version 17; StataCorp) at enrollment. The intervention lasted for 12 months, and assessments were made at enrollment and when the infants were aged 2, 4, 6, 8, 10, and 12 months. Recruitment ran from August 2022 to June 2024. The full details of the PLAY study are described elsewhere [19]. Regarding this substudy, a health literacy intervention was then implemented with the PLAY study intervention group from when the infants were aged 6 months, which constituted enrollment for this substudy. Exit assessments were done at 12 months. Follow-up took place at 8 and 10 months. All health literacy intervention content and delivery methods were initially developed and piloted with a community advisory group (CAG), and examples of the developed content were found to be acceptable [21].

### Ethical Considerations

Ethics approval was obtained from the University of the Witwatersrand Human Research Ethics Committee (substudy M220886 under main study M210846 and M220217). Participant information forms were provided to each participant to read, discuss with, and have any questions answered verbally by a research assistant (RA). Written informed consent was then obtained from all participants for taking part in the focus group discussions (FGDs) and questionnaires and for each FGD to be recorded. Once enrolled in the study, participants were given a unique trial identification number, which was used throughout the study to protect participant privacy and confidentiality. Participant identifier information was kept in a separate project database to maintain anonymity. All participants were reimbursed for travel costs and provided with refreshments at FGDs and hospital-based follow-ups.

### Study Design

**Figure 1:**
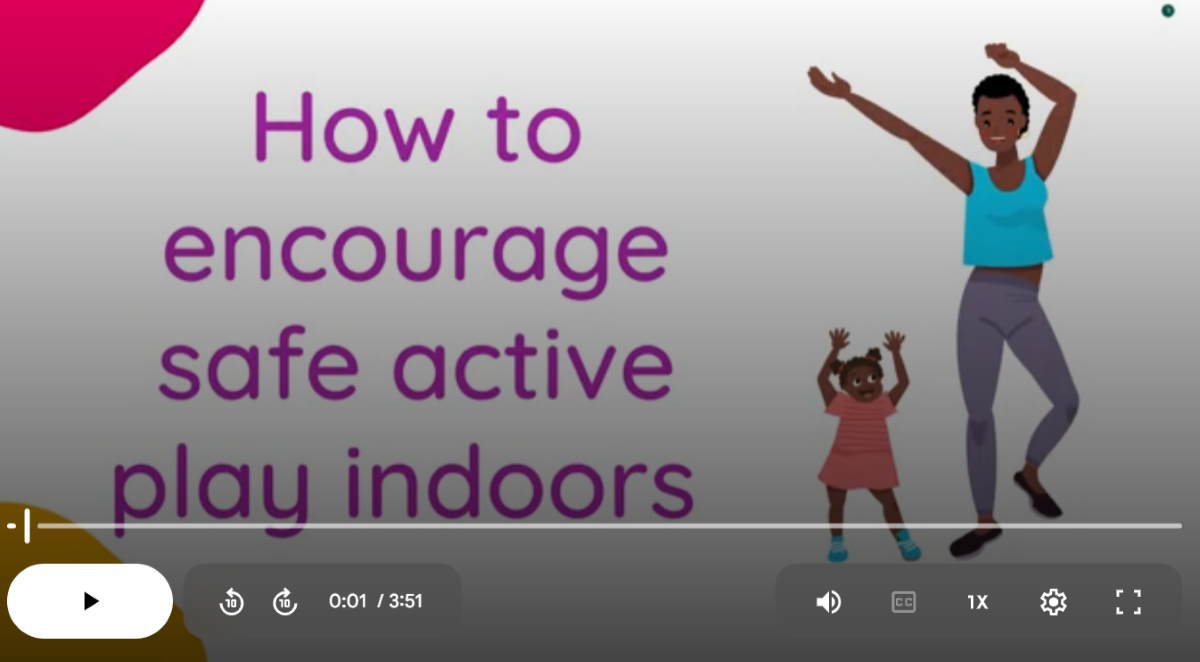
Screenshot of an example of app health literacy video content.

**Figure 2:**
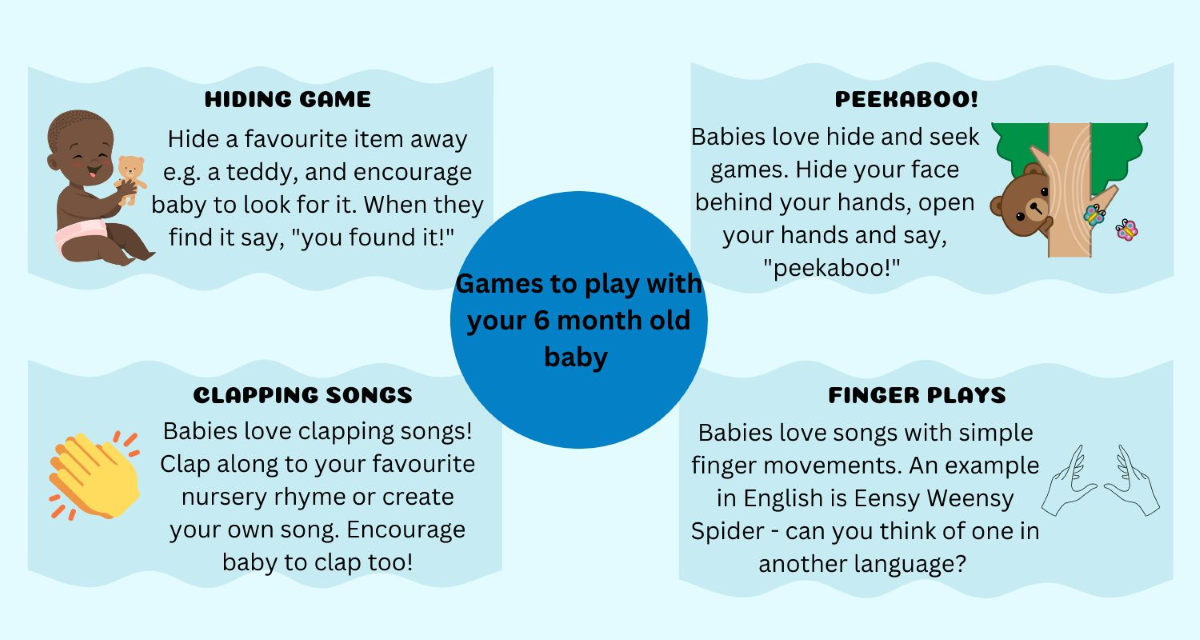
Screenshot of an example of app health literacy infographic content.

This substudy used mixed methods to describe and explore the acceptability and feasibility of the health literacy intervention among the PLAY study intervention group between 6 and 12 months. Mothers randomly assigned to the intervention group (n=68) received telephone or in-person check-ins and assessments conducted at 6, 8, and 10 months and referrals to health care facilities and community services if necessary (standard of care) [19], as well as the intervention content and resources delivered via an HTML5 mobile app deployed to run on the participants’ standard-size smartphones. The app was downloaded and set up on the participants’ smartphones at enrollment. Content was updated weekly so that each participant received content specific to their children’ ages (based on age in months). The health literacy content included infographics and videos showing examples of toys that could be made from recycled material at home, as well as age-appropriate activities and games for infants to encourage active play and development. Informative content such as monthly developmental milestones and information and suggestions regarding safe active play in the home were included. Examples of the video and infographic content are shown in Figures 1 and 2, respectively. This content was codeveloped with the CAG and designed to be specifically contextually relevant for this community [21]. All infographics and video content were provided in English based on CAG recommendations [21]. The initial in-person appointment and enrollment provided a general check-in with the participants to discuss the content and assist with any technical difficulties they may have had when accessing the content through the app. At the 8- and 10-month appointments, specific questions were asked about the infant’s play and development, and the content was discussed again.

### Data Collection

#### Quantitative Data

##### Overview

All quantitative data were collected by the PLAY study team’s RAs using the REDCap (Research Electronic Data Capture; Vanderbilt University) software. The participants’ age, educational level, and income status were collected via a questionnaire at initial PLAY study enrollment (within 1 month of delivery). The acceptability questionnaire was administered to all intervention group participants as part of the 12-month exit assessment from October 2023 to June 2024 by an independent RA to reduce bias. All participants who could not attend in-person 12-month exit assessments were traced and followed up on in their homes.

##### Feasibility

Participant contact (adherence) data were extracted from REDCap to determine the number of participants who attended the 6-, 8-, 10-, and 12-month appointments. As attrition of >20% has the potential to introduce significant bias to the study findings, the intervention was considered feasible if 80% of those participants enrolled in the substudy at 6 months attended the 12-month exit appointment [22]. Finally, questionnaire data on compliance were used to determine how many participants could access content via the app and how frequently they accessed content over the full 12 months of the PLAY study. As this could not be disaggregated to include only access to content from 6 to 12 months, the feasibility of this substudy alone could not be accurately measured using these data, which were included for interest and discussion.

##### Acceptability

**Figure 3:**
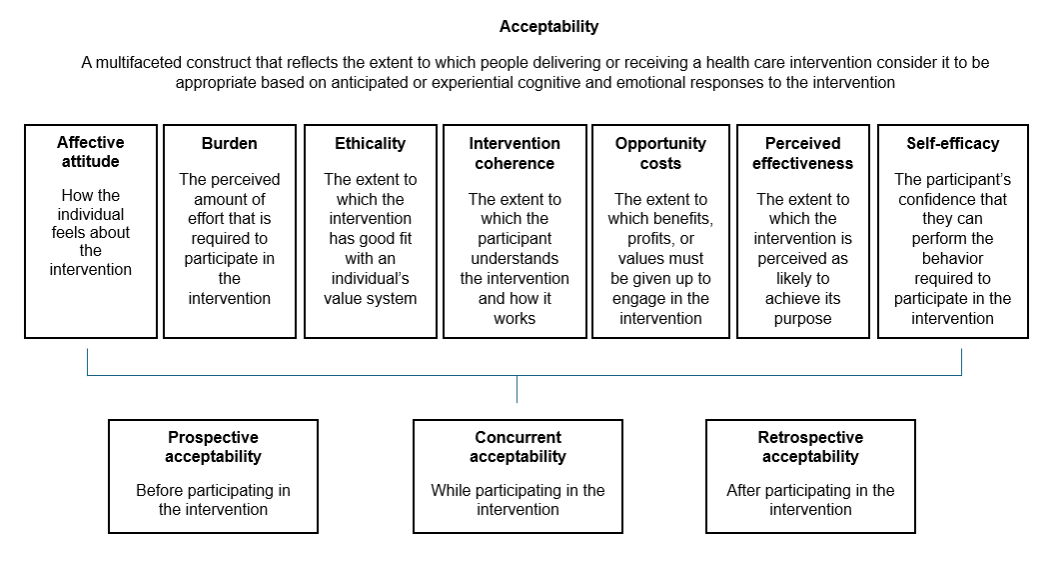
The theoretical framework of acceptability comprising 7 component constructs by Sekhon et al [18].

The acceptability questionnaire, consisting of 22 questions, was compiled by the study team and included questions that captured the acceptability of the health literacy intervention using an ordinal scale. This questionnaire focused specifically on health literacy acceptability to distinguish it from the overall acceptability of the larger study. The acceptability questions were based on the constructs developed by Sekhon et al [18], namely, affective attitude, burden, ethicality, intervention coherence, opportunity costs, perceived effectiveness, and self-efficacy. These constructs were also used to inform the development of the intervention content and, therefore, allowed for continuity of measuring acceptability before and after the study [18,21]. Figure 3 [18] provides a more detailed description of each construct.

#### Qualitative Data

Two qualitative FGDs were conducted by experienced RAs in December 2023 (FGD 1; n=8) and February 2024 (FGD 2; n=9) with intervention group participants who had completed the intervention (n=17). Participants who had completed the acceptability questionnaire by December 2023 were invited to attend FGD 1. This was repeated again for FGD 2; however, those attending the second FGD were required to have also attended the 10-month visit by February 2024 to ensure that we could assess the acceptability of that visit properly.

The FGD guide expanded on the questions asked in the quantitative questionnaire to allow for a better understanding of acceptability of the intervention content. The FGD guide also included questions exploring the participants’ understanding of the 4 study outcome measure questionnaires administered at both 6 and 12 months, namely, the Health Literacy Questionnaire [23], Affordances in the Home Environment for Motor Development–Infant Scale [24], and Assessing Children’s Sedentary and Active Pursuits Questionnaire and Maternal Beliefs Questionnaire [6,25]. The Health Literacy Questionnaire is a standardized questionnaire measuring health literacy according to the following categories: (1) feeling understood and supported by health care providers, (2) having sufficient information to manage the child’s health, (3) actively managing the child’s health, (4) social support for health, (5) appraisal of health information, (6) ability to actively engage with health care providers, (7) navigating the health care system, (8) ability to find good health information, and (9) understanding health information well enough to know what to do [23]. The Affordances in the Home Environment for Motor Development–Infant Scale is an instrument measuring the affordances available for motor development in the child’s environment, asking questions on availability of physical space and variety of stimulation and play materials in the home [24]. The Maternal Beliefs Questionnaire explores the mother’s beliefs, attitudes, and intentions regarding their infant’s physical activity, specifically regarding physical activity knowledge, views on children’s physical activity, physical activity optimism, self-efficacy for promoting physical activity, future expectations regarding the infant’s physical activity and television viewing, and floor play concerns [6,25]. The Assessing Children’s Active and Sedentary Pursuits Questionnaire has two parts: mothers are asked to report (1) the amount of time (minutes) their infant spends in various activities on an average day and (2) whether their child has access to or is likely to have access to age-specific toys and equipment (such as balls, push toys, and bicycles) within the following year [6,25]. As most of the participants were not first-language English speakers and the questions were primarily asked in English (and translated for further clarity if needed), they were asked to discuss any barriers to understanding the questions in each questionnaire. Furthermore, participants were asked to discuss the relevancy of each questionnaire in relation to their context.

Both FGDs were conducted at Chris Hani Baragwanath Hospital in Soweto, Johannesburg, by staff trained in qualitative research data collection. Although questions were asked in English, participants could speak in their home language, and conversations progressed in the vernacular. This was later translated into English (if the response was in a different language) and transcribed for analysis.

### Statistical Analysis

**Figure 4:**
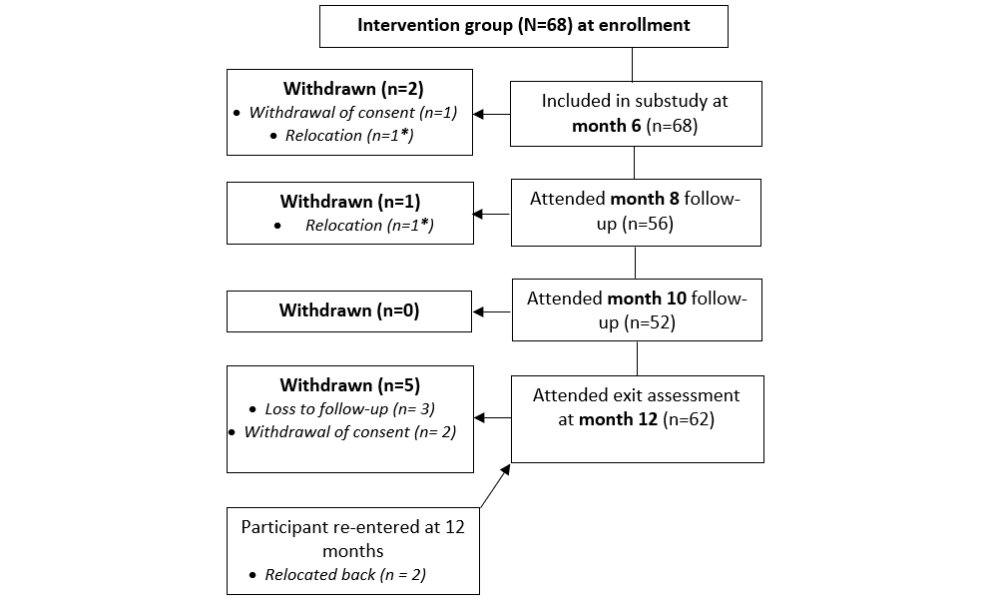
Intervention group attendance (and reasons for withdrawal) at 6-, 8-, 10- and 12- month follow-up appointments.

Descriptive statistics (frequencies and percentages for categorical data and medians and SDs for continuous data) were used to describe the sociodemographic data of the participants, as well as the quantitative acceptability and feasibility data. Similarly, descriptive statistics summarized how many participants attended each appointment and how many participants could access the app. The number of participants who attended the 6-, 8-, 10-, and 12-month follow-up visits was calculated (Figure 4). For each acceptability question, the 2 lowest (1 and 2) and 2 highest (4 and 5) Likert scale scores were combined, resulting in a *low*, *neutral*, or *high* result.

The qualitative data were analyzed by 2 researchers (FB and AP) using thematic network analysis [26]. Meaningful statements and ideas were coded and then grouped into themes, which were summarized and discussed in relation to the original research question. FGD 1 data were coded and classified into loose themes linked to the acceptability constructs. These findings led to the development of the guide for FGD 2, focusing mostly on providing further insights into the themes already established, as well as exploring other themes that were not discussed in depth in FGD 1, such as the validity of the outcome measure questionnaires. The data from FGD 2 were then analyzed using the themes from FGD 1, allowing for new themes to emerge. The codes for the themes from the 2 FGDs were then combined or adjusted accordingly to create new themes and subthemes. At each stage, the analysis was iterative, with researchers double coding the data as well as cross-coding the analyses. When disparities existed, discussions were held, and themes were collapsed or adjusted until agreement was reached.

## Results

### Feasibility

Of the 207 participants screened and invited to take part in the PLAY study, 36 (17.4%) did not complete baseline and randomization, resulting in 171 (82.6%) participants being enrolled in the PLAY study. Of these 171 participants, 87 (50.9%) and 84 (49.1%) were randomized into the intervention group and control group, respectively, after completing the PLAY study enrollment assessment. Of the 87 participants in the PLAY study intervention group, 68 (78%) attended the 6-month assessment, which was the enrollment visit for this substudy. The number of participants who attended the 6-, 8-, 10-, and 12-month follow-up visits for this substudy is summarized in Figure 4.

Participants’ access to the intervention content is shown in Table 1. Most of the participants (47/62, 76%) could access the intervention content over the 12 months of the PLAY study. Of these, 60% (28/47) looked at content more than once a week, and 11% (5/47) looked at it every day, yet just under a quarter (10/47, 21%) only looked at the content a few times.

**Table 1:** Intervention group access to intervention content over 12 months (N=47).

Characteristic	Participants, n (%)
Looked at content every day	5 (11)
Looked at content more than once a week	28 (60)
Looked at content more than once a month	4 (9)
Only looked at content a few times or never	10 (21)

However, despite most participants having access to the content, the access issues experienced by some participants were largely linked to issues beyond the researchers’ control, such as relying on external stakeholders to upload content and update participant profiles in a timely manner, as well as issues with the software, which prevented certain videos from being downloaded despite the content being mobile data free. The study RAs tried to overcome content access challenges by (1) attempting to troubleshoot any software issues when physically with the participants at follow-ups and, if not successful, (2) going through any missed content with the participants at follow-up visits.

### Acceptability

**Figure 5:**
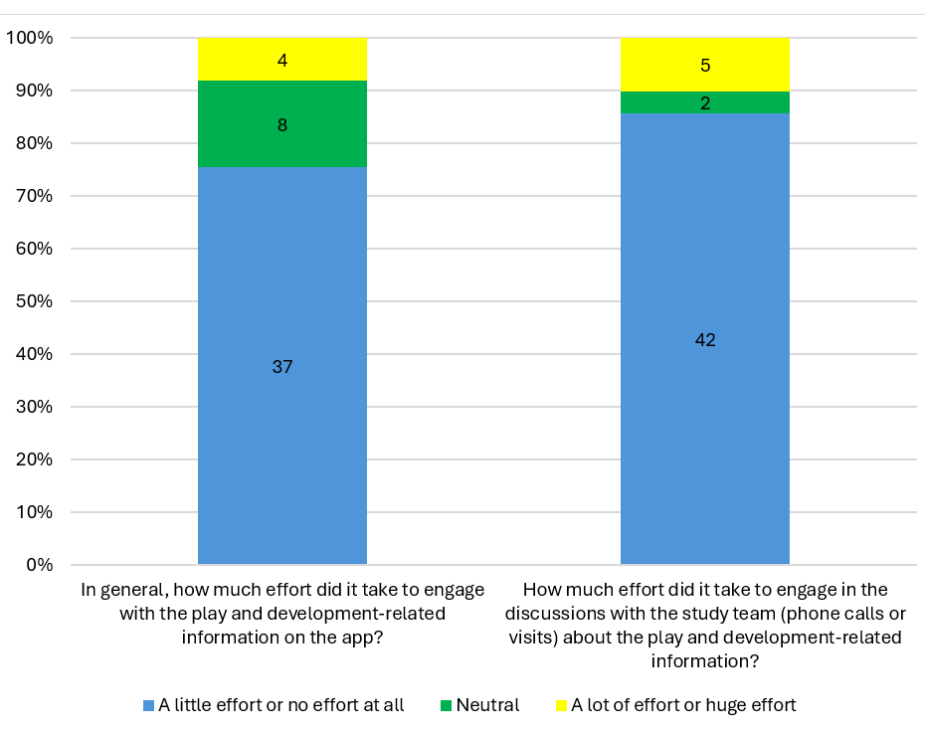
Acceptability questionnaire findings for Burden category in intervention group (N=49).

Of the 62 participants who attended the 12-month exit assessment, 49 (79%) completed acceptability questionnaires. The average age of those who completed the acceptability questionnaire was 29 (SD 5.8) years. Multimedia Appendix 1 shows the results of the acceptability questionnaire. Overall, the questions (such as content ethicality), received very positive responses, with most having a 98% positive result. However, the responses regarding the effort it took to engage with the play and development information on the app were not as positive (37/49, 76%; Figure 5). Furthermore, only 51% (25/49) of the participants always used the information presented to them when deciding which activities to do with their infants (Table 2). Reasons for not engaging with the play and development intervention (opportunity cost) were a participant not having enough time (1/49, 2%) and another participant losing their phone (1/49, 2%; Multimedia Appendix 2). Further details regarding affective attitude, intervention coherence, perceived effectiveness and opportunity cost are shown in Multimedia Appendices 1-4.

**Table 2:** Acceptability questionnaire findings for self-efficacy category in intervention group (N=49).

How often did you use the play and development information presented to you when deciding what activities to do with your baby?	Participants, n (%)
Usually or always	25
Sometimes	23
Hardly ever or never	1

### FGD Results

#### Overview

The combined average age of the participants who attended the focus groups was 30 (SD 5.1) years. In total, 13% (8/62) of the participants attended FGD 1, and 15% (9/62) of the participants attended FGD 2. The themes and subthemes that emerged from the FGDs are discussed in the following sections.

#### Affective Attitude

Participants spoke about what they liked or did not like regarding the intervention content, as well as which aspects they enjoyed the most. Overall, the participants particularly liked the content on making toys and promoting play and development, which was presented in both video and infographic format.

#### Making Toys

The participants enjoyed how the content gave them the possibility of their infants having toys even when participants could not afford to buy them. As the content showed the participants how to make toys out of recycled material (eg, a shaker out of a toilet roll holder and rice), they appreciated that the toys were low cost and easy to make:

It was exciting because we got to learn how to create toys out of nothing. I learned how to make a shaker for a child using a bottle with beads or sand or maybe using eggshells. That turned out to be something that the child can use to shake. We got to know that if we are not able to afford buying toys, we can make them ourselves.

FGD 2

It could be a box and maybe coke plastic bottle and then they would help demonstrate how to create your own toy instead of going to buy one. They were accommodating those who do not have money so that they can also be able to make the choice themselves.

FGD 1

We are all going to say the same things. What I like the most about the app is about making the toys.

FGD 2

Participants expressed enjoying the video content, which showed them step-by-step instructions for making certain toys:

I liked the videos because they would teach us on how to make a toy. They would show you what you needed to make that particular toy.

FGD 1

The participants demonstrated a sense of pride in knowing that they received information and ideas from the study, despite one participant talking about being ridiculed by others for using recycled material to make the toys:

...There is another child where I am staying [who] likes buying juices packaged in a cell phone like bottle and then when they are done drinking, they would give me those bottles so I put beads inside and then I burned the opening to close it up and then when the child shakes it would make sounds. The child enjoyed it a lot and people would laugh at me and then I would tell them to not laugh at me because they had no idea where I got the information about making this toy.

FGD 1

#### Promoting Play and Development

Participants enjoyed the content on promoting play and development, which encouraged them to play with their children and engage in activities that promoted development:

I made different colourful shapes and put them for them [the infant] to choose. Whichever shape they would pick I will tell them if maybe it is a circle. I would encourage them to pick whichever shape I would tell them to.

FGD 2

I liked the information especially about how to encourage the child to sit or crawl.

FGD 1

Some participants reported that the content helped them learn about the importance of play and how to spend time actively playing with their children. Singing and dancing activities were particularly popular:

This Play Study has helped me to know that I should have time for my child and play with him. Not just playing to pass time but I can spend maybe one hour and it must be a routine that we do that every day.

FGD 1

I also liked songs such as “head, shoulders, knees and toes.” We would sing while showing them by pointing their hands, shoulder, knees and toes. I would also sing the song in IsiZulu. We usually sing in front of a mirror and it would excite my child even though they were not understanding what it means. Sometimes I would also point at other body parts while telling them what they are.

FGD 2

What I liked the most was when I got to sing for my child and I could see that they would start dancing because they were enjoying it.

FGD 2

#### Burden, Coherence, and Opportunity Cost

For those participants who could access the intervention content, all of them found it easy to access and use, with no one reporting any problems with access and usability:

I think it was user friendly and simple to understand, depending on the information that I was looking for and I was able to access the information. Sometimes I would refer to the YouTube links for the videos.

FGD 1

When discussing how often the participants engaged with the intervention content, many participants reported that there were other competing priorities, such as work and attending to older children, that would limit their time on the app. This resulted in most participants engaging with the content ad hoc, when they had time:

For me it was finding time to go through the app because I go to work and when I get back, I still need to spend time with the children. On weekends I would be busy with house chores. I hardly had time to go into the app and that was my challenge.

FGD 2

It will be sometimes (going through the app) when I am just sitting alone, I would say let me take the phone and go through the app and read the contents.

FGD 2

Furthermore, most participants found the content easy to understand; however, those who struggled were those who had other tasks or children vying for their attention, therefore limiting their ability to concentrate and comprehend the information:

I do not think there is anything I came across that was difficult. Every information that was there was simple for me to understand. If there was information that I did not understand at the end there will be a video or some pictures which would help me to understand better what they were talking about. For each and every information you would read about there would be a picture attached to it explaining to you that this is what we are talking about.

FGD 2

...Most of the time I am left alone at home because children go to school. I would be on the app but my mind would be wandering somewhere. I could be reading and understanding but my mind would not be in the same space...I know that there is a child in the house and have to keep an eye on them but because there are a lot of things going on and there is no one to talk to. I would tell myself that I need to be focused on the child but my mind would not be there.

FGD 2

#### Perceived Effectiveness

Overall, the participants wanted the content to be accessible to all mothers in their community and were positive about the effectiveness of the content, particularly with regard to its impact on their health literacy and their infants’ development.

#### Infant Development

While many of the participants were aware of the major developmental milestones that children should be reaching at certain ages, they found that the intervention content provided examples of activities to assist their infants in reaching these milestones. The intervention content also assisted in knowing what was considered *normal* with regard to their infants’ development. Furthermore, the participants gained understanding of the importance of play for promoting development:

We knew that he can hold your child to help them to learn to stand or walk on their own for a little while but with the app, you become aware of the milestones and how to assist your child reach them.

FGD 1

I think playing with your child helps with their brain development and stimulating them. At first, I would just think that my son is just playing and I did not know that while he is playing, the development process is also taking place.

FGD 1

#### Health Literacy

Many of the participants reported an increase in their own knowledge of play and development and that their immediate and extended families gained more knowledge as a result of engaging with and sharing the intervention content. The participants felt that the alignment with the information provided by the clinics gave validity to the intervention content and made it seem trustworthy:

Another thing that I have found very important is the fact that I can always reference between the study and the road to health card, that I know what I am doing is right, it is not something that was created from nowhere. So, the fact that there is a backup of the information that I have received.

FGD 2

My eldest daughter is 21 years old and she is the one who made those toys because she would also read through the app. She is studying towards teaching qualification, so this was helpful for her as well. She would play most of the games with the child and with the other sibling. The app was not just helping me but all of us at home.

FGD 1

Some of the participants discussed how the intervention content helped develop their self-efficacy and gave them a sense of independence, which was particularly important for those who did not have support from partners or immediate family:

Another thing that I have just picked up from our sister who was speaking now, when you are a first-time mom and you do not have a mother, there are fathers who are hands on and are able to offer you support. But if you do not have that support structure and you know nothing and you have to teach yourself everything, even with me they just showed me once and then I was able to do everything for myself afterwards.

FGD 1

The participants reported that the intervention content was particularly helpful for those who could not read in teaching them activities to encourage their infants to reach their developmental milestones and the average timelines for each milestone:

I would like to add on that part of people who cannot read...If I am a new mother and do not know anything about the baby, the app shows me step by step. They mentioned the developments expected from the child month to month and if they are not doing what they were supposed to be doing they will tell you to do this and this to encourage the baby. It also shows that your baby will do this and that and this is how you should respond as the mother, you do not have to go to the clinic every time.

FGD 2

### Effect of Assessments on Health Literacy

#### Change in Health Literacy and Behavior Following Questionnaires About Play

While the FGDs sought to determine whether the participants understood the questions asked during the study assessments, it was found that just being asked questions about play and development as part of the assessment questionnaires had an impact on the participants’ behavior, thoughts, and decision-making regarding play environments and resources or toys.

The participants found the assessments relatable and often wanted to find out more about the assessment topic after the session was completed. Many learned more about the stages of development and what behavior was appropriate at the different stages from questionnaires about milestone attainment:

I would say the importance of the milestones. That is the one thing that I can take away for 10 months because immediately after you guys called you asking about if the child can see small objects, can you tell to see a pin and so forth. Some of the questions were eye opening because I never used to take notice of that but after the phone call, I realised that there are few milestones that I have not looked into but now the phone call came, and I wanted to see if my child could do those things you were asking us about.

FGD 2

#### Play Environments

Many of the participants reported that the questions encouraged them to reflect on their home and outdoor environments, particularly related to where their infant could play safely. The participants started to think about how their children could move around actively while being supervised and safe depending on their own home circumstances:

You remember those questions where they asked that, when the child is playing outside, is there anything that they can hold onto for support. I did not realise that when the child is playing outside, I need to guard them. They also asked if there are steps and how big they were. Those questions were very eye opening. I was now able to know what things to look out for when the child is playing to know whether they are in a friendly or danger zone.

FGD 2

For me those questions made me feel somehow because she would be asking if I prefer my child playing or carrying them on my back. At that time, I would then think to myself that if I carry my child on my back, he will not be free to play, but if I put him on the floor, he will be free to play whatever he wants to. For me those questions were relevant because I got to understand that I do not need to confine my child in a small space because of being scared that they were get hurt while playing. Eventually the child will grow and go outside to play.

FGD 2

#### Resources or Toys

Many of the participants discussed how the questions encouraged them to think about what types of toys their infants were playing with and when they should play with different types of toys according to their stage of development. Some did not know that they could introduce toys at an early age, whereas others learned more about the quality of toys and how they could impact their infants’ development in different ways:

For me I can say I was not aware at that time because when the questions were asked, I thought my child was still very young to know about toys. I had not even bought him one toy because I thought he was still young; he cannot play with toys but only with us.

FGD 2

It was eye opening in a sense that toys are not the same and they do not offer the same qualities in terms of the child’s development. Like when you are asked about the book, whether the child has a book. My child has a book, and it is called a Peekaboo Book. The book itself ask about animals, who is hiding behind? It flips open, peekaboo and then it will show you that animal. That was eye opening because firstly the child gets to hear the sounds of the peekaboo that you are saying, and the child can see the colours and animals. For me those kinds of toys felt like they were more educational. As much as they were playful, they were more educational. It was better than having a toy that does not do anything, and it is dark, you do not know. Maybe if it was brighter the child would want it...

FGD 2

## Discussion

### Principal Findings

#### Overview

This study aimed to determine the feasibility and acceptability of an intervention intending to improve maternal health literacy regarding play and development and to determine the participants’ understanding and perceived relevance of the study outcome questionnaires. Recent studies have found the provision of content through mobile apps to be successful in supporting and improving maternal health during the peri- and postnatal periods [27-29]. However, there is an increasing awareness that, for mHealth interventions to improve maternal and child health, they need to be accessible, culturally appropriate, and relevant to the social and physical setting [27,30]. This study adds to the emerging body of knowledge exploring the feasibility and acceptability of this type of intervention in a low- to middle-income country [31,32]. While the intervention was found to be acceptable and feasible, some factors such as access to information on the mobile app were less so due to software-specific issues and preference for information in video format.

#### Feasibility

As >80% of the participants (62/68, 91%) attended the 12-month exit assessment, according to our definition, this study was found to be feasible. When exploring the feasibility of the intervention content and delivery, there were several factors that impacted the participants’ ability to access the play and development content on the app linked to issues with the app software and access to mobile data–free content. These barriers are not limited to this study, and similar problems have been reported in existing maternal or child mHealth technologies (using SMS text messages) available in South Africa such as MomConnect [33], which have been reported as helpful but inconsistent in delivery [16]. Other external factors such as phone sharing, losing phones or having phones stolen, and changing of phone numbers were apparent but expected. These are common problems impacting adherence and delivery of mHealth interventions in similar low-resource settings [16,34,35]. Despite these difficulties, more than half (28/47, 60%) of the participants engaged with the content more than once a week. Other mHealth (SMS text message) studies in low-resource settings have found this frequency of content delivery to be acceptable among users [36]. For those who engaged less frequently, other priorities such as work and looking after other children often took preference. This was similarly noted in the prototyping work with the CAG that informed this study [21].

Study follow-up attendance was found to be relatively high despite the decrease observed in the 8- and 10-month follow-ups. Reasons for the low attrition could be attributed to the RAs developing an effective track-and-trace system, where all uncontactable participants were visited at home. From here, the RAs would make contact again with the participants or be provided with the participants’ location or contact details by a family member or neighbor. Using a telephone to conduct the 10-month follow-up could have had an impact on adherence due to the factors regarding phone use discussed previously. Finally, as there was a focus on collecting data for the outcome analysis time points, this led to higher attendance rates at both the 6- and 12-month follow-up time points. It is also important to note that, even though participants may not have attended each follow-up visit, they were still engaging with the weekly content via the app.

#### Acceptability

Overall, there was a high level of acceptability of the intervention among the participants. The least acceptable components of the intervention (that were still considered very highly acceptable at 37/49, 76%) regarded how often the participants used the information when deciding on what activities to do and the effort it took to engage with the information. In the questionnaire, the participants reported that they preferred meeting in person, and no participants reported that they preferred engaging with the infographics. This correlates with findings from the preliminary prototyping work with the CAG, which found that 90% of the group preferred team discussions and videos compared to the infographics when asked in a questionnaire but were more favorable toward the pictures during the discussion [21]. Unfortunately, due to limited resources for producing high-quality videos in this pilot study, the researchers had to provide a combination of videos and infographics to ensure the validity of the information shared. Similarly, while some participants liked reading the content information, some reported that they particularly liked the videos as they gave them step-by-step instructions that were easy to follow. This has also been reported in other low-resource settings where low (health) literacy levels can result in misinformation and limit understanding of written materials such that oral instructions or other traditional communication (eg, radio and television) are deemed more acceptable and feasible [16]. While other exploratory work in a similar setting found that women were skeptical about receiving information that would not elicit any change in behavior due to constraints in their environment [16], participants in this study were very positive about the intervention and the change it could achieve, particularly when used alongside the Road to Health card and information provided by health facilities. This can largely be attributed to the content being so contextually relevant as a result of involving a small group of mothers from this setting (the CAG) in testing the initial prototype, which was then used to create our final intervention content [21]. Furthermore, aligning the content with government-provided information played an important role in increasing the intervention’s acceptability, the participants’ ability to trust the information, and their ability to use it alongside the resources they already had. This has been observed in text-based mobile interventions in a similar context, where the provision of trusted health information resulted in positive health outcomes [37].

The participants found the questionnaires easy to understand, but this was largely impacted by having an RA who was able to translate any difficult words into the participants’ home language if required. While participants found the questionnaires relatable to their context, an interesting finding was how the questionnaires did not just serve as outcome measures but acted as a means of improving knowledge regarding play, development, and the environment. This was largely due to participants being required to consider whether their environments were conducive to their children playing safely and discussing the importance of toy use at different ages to encourage development. This phenomenon is an extension of the Hawthorne effect (which occurs when individuals modify their behavior in response to being observed or knowing that they are part of an experiment), also known as measurement bias or “measurement reactivity” [38]. Measurement bias is commonly observed when repeating the same questionnaires (response shift) and when the act of completing a questionnaire results in new beliefs and behaviors regarding the research topic [39]. However, the Hawthorne effect usually disappears in well-designed RCTs, as would be expected in this study [38].

#### Potential for Effectiveness

The high acceptability of the intervention content and belief that other mothers would benefit from it provides great potential for observing effectiveness in similar communities. As there was such a positive response from the participants regarding being able to make their own toys and learn new skills, in practice, this ability would allow other mothers in similar settings to have access to these resources, therefore encouraging and equipping them to foster more play and, subsequently, further development in their infants. The participants reported an increase in knowledge of infant play and development and self-efficacy after engaging with the content, which supports other studies highlighting a positive association between health literacy and self-efficacy [40,41]. As self-efficacy has been found to be a mediator between maternal health literacy and positive parenting behaviors (ie, encouraging participation in infant play) [8], this links positively to the overall trial study objective of encouraging maternal self-efficacy using different microinterventions. Testing feasibility and acceptability is an important first step as it provides important guidelines when scaling up studies or implementing these interventions in practice.

### Study Strengths and Weaknesses

Using mixed methodology helped improve the validity of the study findings, where insight into the quantitative findings could be challenged or consolidated through the qualitative FGDs. While there were technical issues related to delivering the app in this study (which can be easily rectified), the content of the app and overall intervention were found to be highly acceptable to the participants and feasible in this setting. This study’s main limitations were linked to the feasibility of using inconsistent external service providers to deliver the content via a prototype mobile app, which resulted in technical issues (despite the app being mobile data free). To resolve this, the researchers have created an independent app containing the resource content, which counteracts the issues encountered in this feasibility study and can be implemented at scale. With regard to measuring feasibility, the health literacy intervention was provided from infant age of 6 to 12 months, but the larger study provided other content from 0 to 12 months. Therefore, we could not determine what content was received by each individual and when. However, purposively recruiting FGD participants who had received the entire 6- to 12-month intervention, including app content and 10-month follow-up attendance, allowed us to have a better, more in-depth understanding of the acceptability of the intervention provided specifically during those months. It is important to note that only FGD 2 included participants who had attended the 10-month follow-up visit. An additional FGD with participants who attended the 10-month follow-up may have provided further insight on acceptability from those who received the entire intervention. While it is assumed that most participants used Android phones (due to affordability), this was not documented, which would have been helpful to determine whether there were different user experiences between Apple iOS and Android devices.

### Implications for Future Research and Implementation

These findings suggest that interventions that provide meaningful, clear, and easily accessible information on play and development in infants would be useful and well received by mothers and caregivers in similar settings and show potential for effectiveness in improving health literacy regarding play and development. Given the digital limitations in this population, researchers and policy makers need to develop interventions that provide information through a variety of platforms to enable equitable access for all.

### Conclusions

This study provides insight into the acceptability and feasibility of an intervention aimed at improving maternal health literacy regarding play and development in a low-resource setting. While the intervention was considered feasible within this setting and highly acceptable to participants, barriers related to external management of the app resulted in some issues with accessibility. Importantly, developing the intervention content with the guidance of community members resulted in very high acceptability and potential for effectiveness.

## Appendix

Multimedia Appendix 1 The 12-month acceptability questionnaire (intervention group) detailing affective attitude cost findings (N=49).

Multimedia Appendix 2 The 12-month acceptability questionnaire (intervention group) detailing intervention coherence findings (N=49).

Multimedia Appendix 3 The 12-month acceptability questionnaire (intervention group) detailing perceived effectiveness findings (N=49).

Multimedia Appendix 4 The 12-month acceptability questionnaire (intervention group) detailing opportunity cost findings (N=49).

## Abbreviations

CAG: community advisory group
FGD: focus group discussion
mHealth: mobile health
PLAY: Play Love and You
RA: research assistant
RCT: randomized controlled trial
REDCap: Research Electronic Data Capture

## Data Availability

The datasets generated or analyzed during this study are available from the corresponding author on reasonable request.
