# Supplementary figures and images for: The Feasibility and Acceptability of an App-Based Intervention Aimed at Improving Maternal Health Literacy Regarding Infant Play and Development: Mixed Methods Study
Source: JMIR Form Res. 2025 Sep 4;9:e76517. doi: 10.2196/76517 (PMC12447011; doi:10.2196/76517)

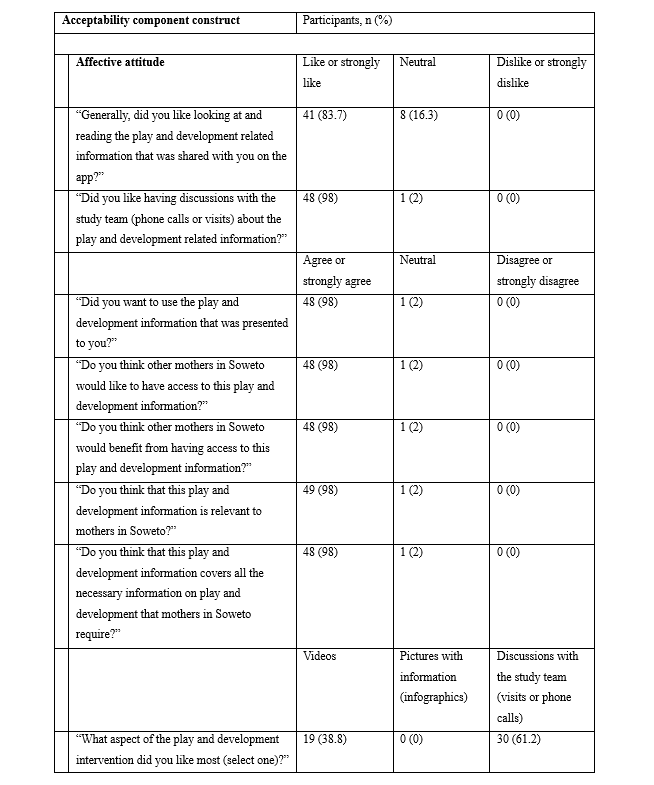

Supplement: Multimedia Appendix 1 [file formative_v9i1e76517_app1.png]

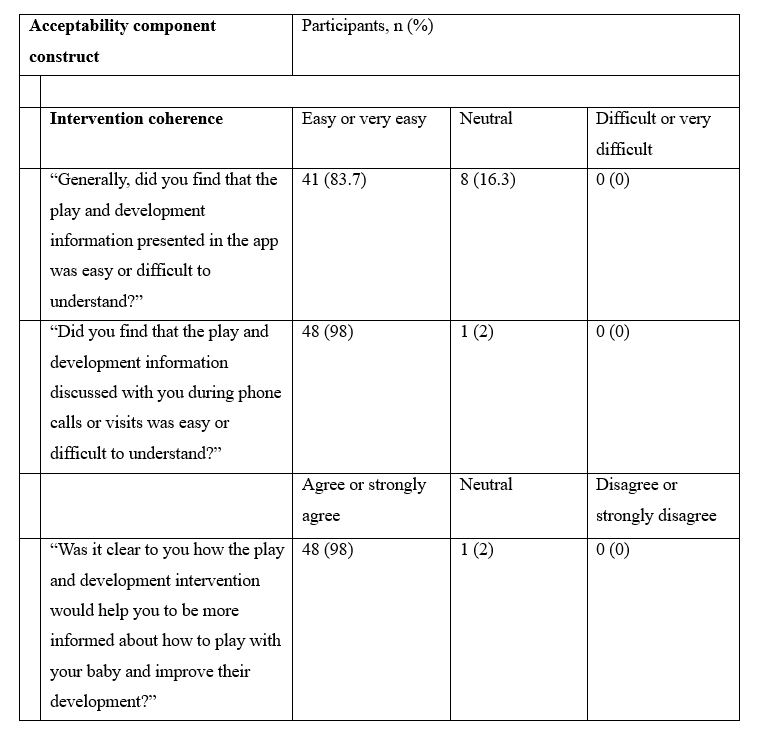

Supplement: Multimedia Appendix 2 [file formative_v9i1e76517_app2.png]

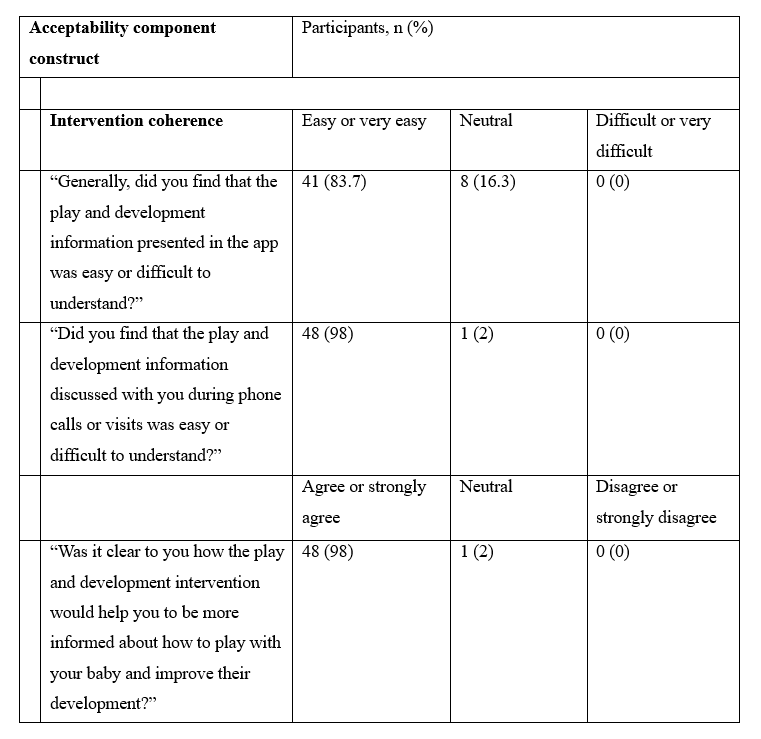

Supplement: Multimedia Appendix 3 [file formative_v9i1e76517_app3.png]

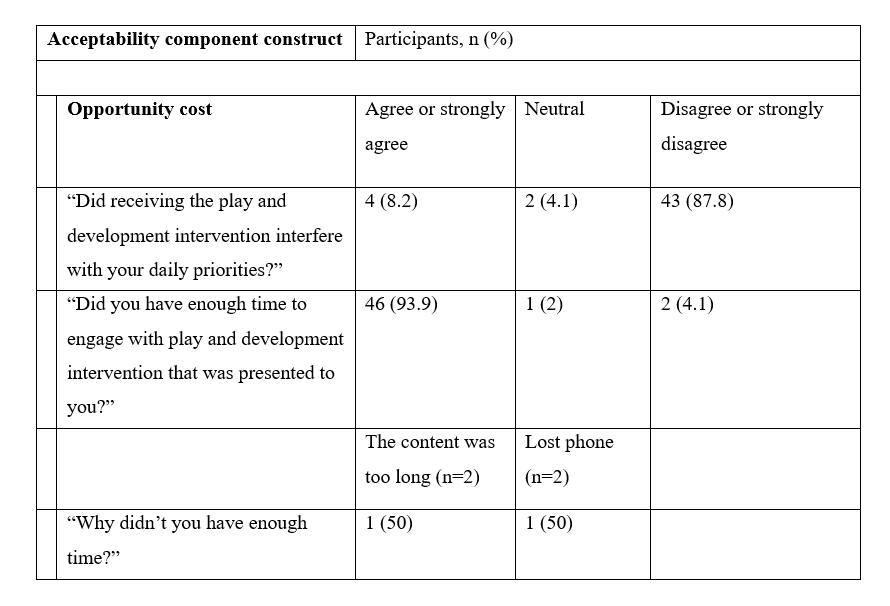

Supplement: Multimedia Appendix 4 [file formative_v9i1e76517_app4.png]
